# Increased tumour dihydroceramide production after Photofrin-PDT alone and improved tumour response after the combination with the ceramide analogue LCL29. Evidence from mouse squamous cell carcinomas

**DOI:** 10.1038/sj.bjc.6604896

**Published:** 2009-02-17

**Authors:** D Separovic, J Bielawski, J S Pierce, S Merchant, A L Tarca, B Ogretmen, M Korbelik

**Affiliations:** 1 Department of Pharmaceutical Sciences, Eugene Applebaum College of Pharmacy and Health Sciences, Wayne State University, 259 Mack Ave., Detroit, MI 48201, USA; 2 Karmanos Cancer Institute, 4100 John R, Wayne State University, Detroit, MI 48201, USA; 3 Department of Biochemistry and Molecular Biology, Medical University of South Carolina, 173 Ashley Ave, Charleston, SC 29425, USA; 4 Department of Cancer Imaging, British Columbia Cancer Agency, 675 West 10th Ave., Vancouver, British Columbia V5Z 1L3, Canada; 5 Department of Computer Science, Wayne State University, 5143 Cass Ave., Detroit, MI 48202, USA

**Keywords:** C6-pyridinium ceramide, dihydroceramide, plasma, PDT, Photofrin, tumour

## Abstract

Photodynamic therapy (PDT) has been proven effective for treatment of several types of cancer. Photodynamic therapy alone, however, attains limited cures with some tumours and there is need for its improved efficacy in such cases. Sphingolipid (SL) analogues can promote tumour response in combination with anticancer drugs. In this study, we used mouse SCCVII squamous cell carcinoma tumours to determine the impact of Photofrin-PDT on the *in vivo* SL profile and the effect of LCL29, a C6-pyridinium ceramide, on PDT tumour response. Following PDT, the levels of dihydroceramides (DHceramides), in particular C20-DHceramide, were elevated in tumours. Similarly, increases in DHceramides, in addition to C20:1-ceramide, were found in PDT-treated SCCVII cells. These findings indicate the importance of the *de novo* ceramide pathway in Photofrin-PDT response not only in cells but also *in vivo*. Notably, co-exposure of SCCVII tumours to Photofrin-PDT and LCL29 led to enhanced tumour response compared with PDT alone. Thus, we show for the first time that Photofrin-PDT has a distinct signature effect on the SL profile *in vitro* and *in vivo*, and that the combined treatment advances PDT therapeutic gain, implying translational significance of the combination.

Ceramide mimetics and drugs targeting sphingolipid (SL) metabolism have made major advances towards cancer treatment (Fox *et al*, 2006; Zeidan and Hannun, 2007). To overcome the low solubility of ceramide, cationic pyridinium ceramide analogues have been developed, which are water soluble (Szulc *et al*, 2006). These analogues have effective anticancer activity at relatively low concentrations (Novgorodov *et al*, 2005; Rossi *et al*, 2005; Dindo *et al*, 2006; Senkal *et al*, 2006; Zeidan and Hannun, 2007; Dahm *et al*, 2008). LCL124, alone or in combination with the chemotherapeutic agent gemcitabine, inhibits substantially the growth of human head and neck squamous cell carcinomas *in vitro* and *in vivo* (Senkal *et al*, 2006). LCL30 has potent antitumour activity against colorectal cancer in mice (Dahm *et al*, 2008).

The oxidative stress inducer photodynamic therapy (PDT) uses a photosensitiser, visible light and oxygen to generate reactive oxygen species that can destroy malignant cells by apoptosis (Dougherty *et al*, 1998; Miller *et al*, 2007). We have shown in Jurkat cells with downregulated sphingomyelin synthase that an enhanced accumulation of ceramide and dihydroceramide (DHceramide) correlates with the promotion of apoptosis post-Pc 4-PDT (Separovic *et al*, 2008). We have also demonstrated that the combination of Pc 4-PDT with exogenous C16-ceramide increases mitochondrial depolarisation and apoptosis in Jurkat cells (Dolgachev *et al*, 2003). There is need to verify the relevance of our cell culture findings in tumour models. A novel effective ceramide mimetic LCL29 (Szulc *et al*, 2006; Bielawska *et al*, 2008), a structural analogue of LCL124, was used to test its ability to enhance the response of mouse SCCVII squamous cell carcinomas to Photofrin-PDT. The photosensitiser Photofrin was used because of its clinical relevance as it is the only photosensitiser approved by the Food and Drug Administration for cancer treatment in the United States. The main objectives of this study were (i) to determine signature effects of Photofrin-PDT on endogenous ceramide and DHceramide in SCCVII cells and tumours and (ii) to determine the therapeutic effect of the combination PDT+LCL29 in SCCVII tumours.

## Materials and methods

### Tumour-related cell model and PDT treatment

As shown earlier (Cecic and Korbelik, 2006), mouse SCCVII squamous carcinoma cells were grown in Alpha Minimal Essential Medium (Sigma-Aldrich, St Louis, MO, USA) containing 10% heat-inactivated foetal bovine serum (Hyclone, Logan, UT, USA). The photosensitiser Photofrin (Axcan Pharma, Mont-Saint-Hilaire, QC, Canada) was dissolved in 5% dextrose. For the experiment, following overnight incubation with Photofrin (20 *μ*l ml^−1^), cells (4.5 × 10^6^) were irradiated at 0.5 or 1 mJ cm^−2^ using a beam of 630±10 nm. To generate the beam, a 600-W quartz-halogen light source with infrared radiation reduced by a 10-cm layer of water and 850-nm cutoff filter was used. The bandwidth was further confined to 630±10 nm by a narrowband-interference filter (Oriel Instruments, Stratford, CT, USA). Two or four hours after PDT, cells were collected for mass spectrometry (MS).

### Tumour model and PDT treatment

As described earlier (Sun *et al*, 2002), female C3H/HeN mice were implanted with syngeneic SCCVII squamous cell carcinoma tumours (Khurana *et al*, 2001) by a subcutaneous injection of 1 × 10^6^ SCCVII cells at a lower dorsal site. After 7–8 days, the tumours reached a size of 6–8 mm in largest diameter. At that point treatment began. The cohort of SCCVII tumour-bearing mice was divided into groups for various treatments. Tumour growth was monitored and recorded by measuring three orthogonal tumour diameters (*a*, *b* and *c*) using a caliper. Tumour volume (*V*) was calculated using the formula: *V*=Π × ab × *c*/6. The measurement was taken every second day. Mice were killed before starting to suffer from tumour burden, that is, at the point when tumour size was 15 mm in diameter. For PDT, tumours were treated 24 h after photosensitiser administration with light produced by an FB-QTH high-throughput illuminator (Sciencetech, London, ON, Canada). Light was delivered through a 630±10-nm interference filter for superficial tumour illumination with a fluence rate of 80–90 mW cm^−2^. During treatment of tumours with light, the mice were immobilized with metal holders. Thereafter, the mice were monitored up to 14 days post-therapy and the presence of tumours or their absence recorded every 2 days. The ceramide analogue D-erythro-2-*N*-[6′-(1′′-pyridinium)-hexanoyl]-sphingosine bromide (LCL29, also known as C6-pyridinium ceramide) was obtained from Avanti Polar Lipids (Alabaster, AL, USA). For experiments, LCL29 was dissolved in distilled water and injected intraperitoneally (20 mg kg^−1^) daily over a period of 7 days. The first injection was administered 2 h before PDT light treatment. Blood obtained from mice by cardiac puncture was collected into EDTA-containing tubes, and plasma samples together with samples of tumour tissue homogenates were analysed for the SL profile using MS. The procedures with mice were approved and overseen by the Animal Care Committee of the University of British Columbia.

### Measurement of SLs by electrospray ionisation/double MS

As we have shown earlier (Separovic *et al*, 2007; Separovic *et al*, 2008) following extraction, SLs were separated by high-performance liquid chromatography, introduced to electrospray ionisation source and then analysed by double MS using TSQ 7000 triple quadrupole mass spectrometre (Thermo-Fisher Scientific, San Jose, CA, USA) which allows the simultaneous determination of various SLs, including various ceramide and DHceramide species, dihydrosphingosine (DHsphingosine), sphingosine and sphingosine-1-phosphate (S1P) (Bielawski *et al*, 2006). Specifically, samples obtained from cells or tissues were fortified with the internal standards (C17-base-D-e-sphingosine, C17-sphingosine-1-phosphate, *N*-palmitoyl-D-e-C13-sphingosine and C17-D-e-sphingosine) and extracted with ethyl acetate/isopropanol/water (60/30/10, v/v). After evaporation and reconstitution in methanol, samples were injected into the HP1100/TSQ 7000 LC/MS system and gradient eluted from the BDS Hypersil C8, 150 × 3.2 mm, 3-*μ*m particle size column, with 1 mM methanolic ammonium formate/2 mM aqueous ammonium formate mobile phase. Peaks corresponding to the target analytes and internal standards were collected and processed using the Xcalibur software system. Quantitative analysis is based on the calibration curves generated by spiking an artificial matrix with known amounts of the target analyte synthetic standards and an equal amount of the internal standards. For the calibration curves, the target analyte/internal standard peak area ratios are plotted against analyte concentrations. The target analyte/internal standard peak area ratios from the samples are similarly normalised to their respective internal standards and compared with the calibration curves, using a linear regression model.

### Statistical analysis

For statistical analysis of MS data, unless indicated otherwise, the mass of SLs in pmol mg^−1^ was log2 transformed to improve the normality of the distributions and allow computation of more robust means and standard deviations. As 26–29 SLs were measured and tested for differences at the same time, besides nominal *P*-values obtained by *t*-test, adjusted *P*-values (*P*<0.1) using the false discovery rate (FDR) method were used to infer significance (Benjamini and Hochberg, 1995). The R statistical environment (www.r-project.org) was used for all computations. For *in vitro* studies, the analysis of the interaction between Photofrin and light dose, in addition to assessing the effect of time, was performed by linear models (Wilkinson and Rogers, 1973). For MS *in vivo* studies, a two-tailed *t*-test was employed to compare concentration levels of SLs between treatment groups, as well as to test whether fold changes for the entire family of SLs (e.g., ceramides) were different than 1. To assess the effect of the combined treatment on tumour reduction, a linear mixed-effects model was used (Pinheiro and Bates, 2000).

## Results and discussion

### Signature effects of Photofrin-PDT on the SL profile of *in vitro* cultured SCCVII cells

**Figure 1:**
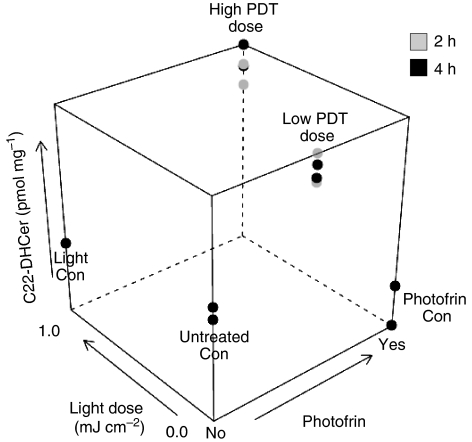
Photofrin-PDT increases C22-DHceramide in SCCVII cells. After overnight incubation with Photofrin (20 *μ*g ml^−1^), cells were irradiated at 0.5 (low) or 1 mJ cm^−2^ (high) light dose, incubated at 37°C for 2 or 4 h and collected for MS analysis. Con, corresponding controls. A three-dimensional plot of C22-DHceramide levels as a function of treatment conditions is shown. Unlike Photofrin or light alone, PDT triggered a significant increase in C22-DHceramide levels.

SCCVII cells were exposed to low and high PDT doses corresponding to ∼LD_30_ and LD_80_, respectively (Cecic and Korbelik, 2006), incubated for 2 or 4 h at 37°C, and collected for MS. Neither Photofrin nor light alone had significant effects on basal levels of SLs (not shown). Dose responses of SLs to PDT were statistically evaluated against their corresponding controls. The levels of C20:1-ceramide, C14-, C16-, C18:1- and C22-DHceramide significantly rose to 7.3-, 10.5-, 11.8-, 9.6- and 5.3-fold, respectively, by 4 h per 1 mJ cm^−2^ of light fluence (Table 1). A dose-dependent increase in C22-DHceramide is shown in Figure 1. Globally, the levels of ceramides and DHceramides significantly increased to 4.3- and 5.0-fold of their corresponding controls per 1 mJ cm^−2^ of light fluence (not shown). Thus, there was global and selective increase in ceramides and DHceramides per unit of PDT light dose.

The levels of other SLs were also evaluated with respect to PDT dose. DHsphingosine-1-phosphate was increased 9.8-fold per unit of PDT dose (Table 1). Although the nominal *P*-value indicates a significant change, the chosen FDR value does not, which could be a false-negative result. There was no significant effect of PDT dose on the levels of DHsphingosine, sphingosine or S1P (Table 1).

Time dependence of the SL response to PDT was also analysed. Individual ceramides did not show significant time-dependent changes post-PDT. However, there was a significant, 12.5% average increase per hour post-PDT in the levels of all 11 ceramides. The chance that this was a random event is only 0.00048 (not shown). Other SLs, including DHceramides, individually or globally, did not show significant changes over time post-PDT (not shown).

Overall, these are important findings because they demonstrate that the accumulation of ceramide and DHceramide is not only limited to Pc 4-PDT, as we have shown earlier (Separovic *et al*, 2007; Separovic *et al*, 2008), but can also be evoked after Photofrin-PDT. These data also demonstrate the involvement of the *de novo* ceramide pathway after photodamage.

### *In vivo* basal SL profile shows the prevalence of ceramides

**Figure 2:**
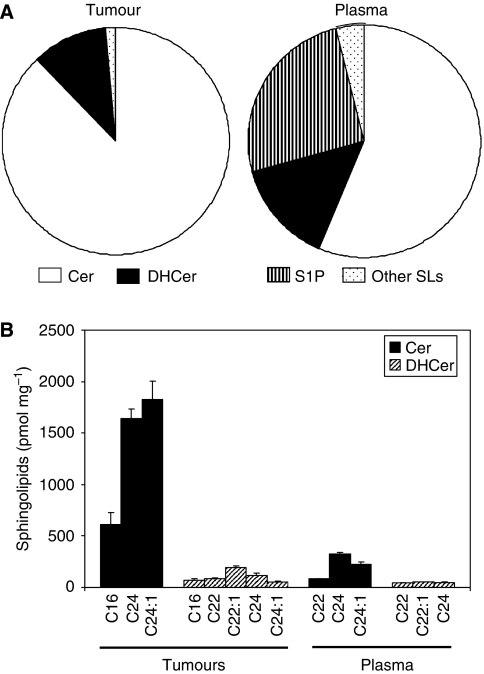
SL levels in untreated SCCVII tumours and host plasma. Using cardiac puncture, blood was collected into EDTA tubes. Tumours were excised and their homogenates as well as plasma samples were processed for MS analysis. (**A**) Each SL shown as the percentage of total SLs in tumours and plasma, respectively. (**B**) The levels of most abundant ceramides and DHceramides (pmol mg^−1^) in untreated SCCVII tumours and host plasma. The data for each SL are expressed as the mean±s.e.m. (*n*=3–4).

Ceramides and DHceramides detected in untreated SCCVII tumours comprised 89 and 11% of the total SL mass, respectively (Figure 2A). In the plasma of mice bearing these tumours, ceramides, DHceramides and S1P comprised 56, 14 and 25% of the total SL mass at rest, respectively (Figure 2A). Virtually all S1P was found in the plasma. Vascular endothelium has been suggested to contribute to mouse plasma S1P (Venkataraman *et al*, 2008).

The levels of ceramides and DHceramides that comprise 10% or more of the total corresponding SLs are depicted in Figure 2B. The most abundant ceramides in growing untreated SCCVII tumours were C16-, C24- and C24:1-ceramide comprising 13, 36 and 40% of total ceramides, respectively (Figure 2B). Interestingly, C16-ceramide, not C24:1-ceramide, is the most abundant ceramide in human head and neck squamous carcinomas (Koybasi *et al*, 2004). One reason for the discrepancy could be that non-tumour cells associated with these tumours are different.

In plasma, the levels of C16-ceramide were restricted below 1% of total plasma ceramides. Plasma C24-ceramide comprised nearly 50% of total ceramides. Comparative levels of tumour and plasma C16-DHceramide followed the same pattern as C16-ceramide (Figure 2B). The very-long fatty acyl chain DHceramides, C22-, C22:1- and C24-DHceramide were among the most abundant in both tumours and plasma. Overall, ceramides and DHceramides with long and very-long fatty acyl chains were the most abundant species in these tissues.

### Global increase in tumour DHceramides after Photofrin-PDT

**Figure 3:**
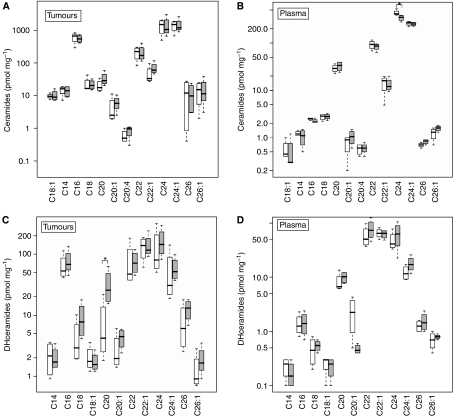
Mass spectrometric analysis reveals distinct signature effects on the *in vivo* SL profile after Photofrin-PDT. (**A** and **B**) The levels of tumour and plasma ceramides, respectively. (**C** and **D**) The levels of tumour and plasma DHceramides, respectively. Photofrin (10 mg kg^−1^) was injected i.p. 1 day before exposure of SCCVII-bearing mice to light (150 J cm^−2^). Four hours after PDT, mice were killed. For other details, see Figure 2. Each experimental group consisted of four mice. In the plots, for *y* axis, log scale is used. The boxes contain 50% of the data and the median value is shown as the horizontal thick line within the box. The whiskers extend to the most extreme data point (minimum and maximum value), which is not more than 1.5 times the interquartile range (i.e., remaining 50% of the data) from the box. Significance (*P*<0.05) between Photofrin alone and PDT for a particular SL molecule was determined by *t*-test and is indicated by an asterisk. Empty boxes, Photofrin alone; filled boxes, Photofrin-PDT.

SCCVII tumours were treated with a therapeutic dose of Photofrin-PDT (Korbelik and Cecic, 2008). Tumour and plasma SL profiles were identified by MS (Figure 3). Among plasma ceramides, only the levels of C24-ceramide were 29% lower after PDT compared with Photofrin alone (Figure 3B). Notably, following PDT the levels of tumour C20-DHceramide increased to 5.4-fold of Photofrin alone (Figure 3C). There were no significant differences in the levels of specific tumour ceramides or plasma DHceramides after PDT compared to Photofrin alone (Figures 3A and D).

**Figure 4:**
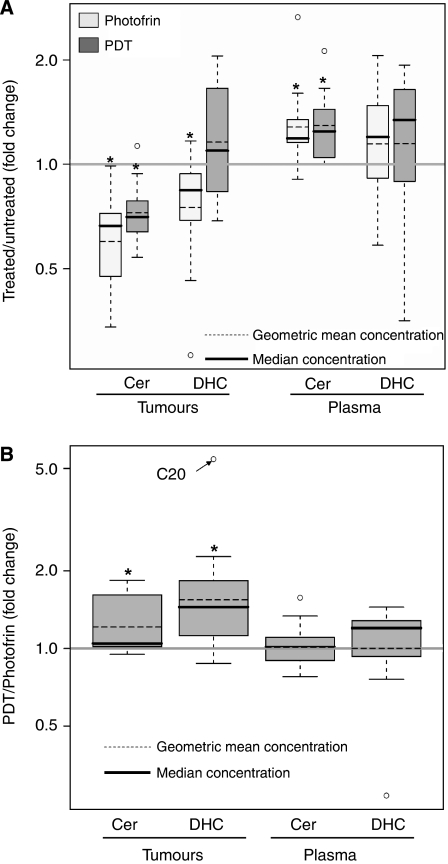
Global increase in tumour DHceramides after Photofrin-PDT. Total fold changes were calculated for averages of all ceramides or DHceramides after PDT relative to averages of all corresponding SLs in untreated (**A**) or Photofrin-treated mice (**B**). Significance (*P*<0.05) between PDT and untreated or Photofrin alone for total ceramides and DHceramides was determined by *t*-test and is indicated by asterisks. Outliers are shown as empty circles. In (**B**), the fold change for tumour C20-DHceramide is shown as an outlier and pointed by an arrow (cf. Figure 3C). For other details, see Figure 3. The horizontal line at a value of 1.0 indicates no change. Cer, ceramides; DHC, DHceramides.

In addition, overall fold changes were calculated for averages of all ceramides or DHceramides after PDT relative to averages of all corresponding SLs in untreated or Photofrin-treated mice. Compared with untreated controls, the levels of total tumour ceramides were reduced by 67 and 38% after Photofrin and PDT, respectively (Figure 4A). Compared with Photofrin, a 21% attenuation of tumour ceramide response to PDT was significant (Figure 4B). A significant 1.3-fold total increase in plasma ceramides was observed following either Photofrin alone or PDT (Figures 4A and B).

Relative to untreated controls, a 34% total decrease in tumour DHceramides was observed post-Photofrin (Figure 4A). In contrast, exposure of mice to PDT led to a 15% total increase in tumour DHceramides relative to untreated controls (Figure 4A). Thus, compared with Photofrin alone, there was an overall significant 1.6-fold increase in tumour DHceramides post-PDT (Figure 4B). Total increases in plasma DHceramides were similar in mice exposed to either treatment (Figures 4A and B).

No significant effect on tumour S1P levels was observed after PDT. In plasma, Photofrin alone or PDT decreased S1P levels by 31% so that there was no difference between the two (not shown). Neither tumour nor plasma sphingosine levels were significantly affected by PDT (not shown).

In summary, these are significant data not only because they confirm our cell culture findings but also because they show for the first time that a therapeutic dose of Photofrin-PDT triggers a significant, potentially selective, build-up in tumour DHceramides, supporting the involvement of the *de novo* ceramide pathway in PDT *in vivo* response. Photofrin alone also affects the SL profile, but the impact of PDT is distinctly different, particularly in triggering more dramatic increases in DHceramide levels. The effects of photosensitiser alone on SLs are expected to be less pronounced, with more potent sensitising agents administered at much lower doses than Photofrin.

### Enhanced tumour response after Photofrin-PDT+ceramide analogue LCL29

**Figure 5:**
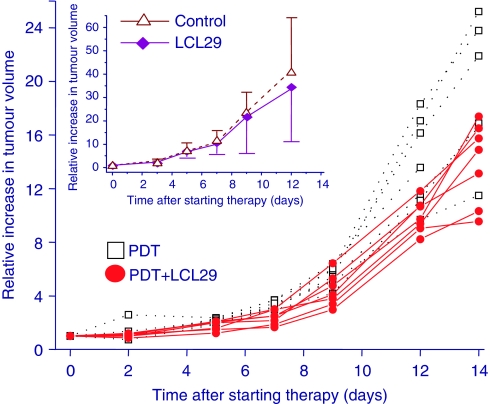
Enhanced response of SCCVII tumours to Photofrin-PDT+LCL29. SCCVII tumours were treated with PDT (Photofrin 5 mg kg^−1^ plus 150 J cm^−2^ 24 h later), LCL29 (seven daily injections of 20 mg kg^−1^ i.p.) or their combination (initial LCL29 injection 2 h before PDT light was followed by additional six daily injections). A control group and PDT-only group received vehicle injections as in the LCL29 treatment protocol. The therapy response was monitored by tumour size measurement. Depicted in the insert are the results (presented as the means±s.d.) for LCL29 treatment alone and the control group with vehicle i.p. injections. The results in the main graph are presented as separate growth curves for individual tumours. Tumours in PDT+LCL29 group grew slower than the majority of those in PDT-only group. On the basis of the linear mixed-effects model, the difference between these two groups is significant with *P*<0.012 (*n*=7). Volume normalisation was performed by dividing the tumour volume data by the tumour volume measured on day 0.

To test whether the ceramide analogue LCL29 can improve PDT tumour response, SCCVII tumours were treated with a moderately therapeutic dose of Photofrin-PDT+non-toxic dose of LCL29 (Senkal *et al*, 2006). LCL29 was administered daily over 7 days, starting as the combination with PDT. As shown in Figure 5 (insert), LCL29 alone had no effect on tumour growth. Remarkably, growth retardation of mouse tumours attained by PDT was enhanced by adjuvant LCL29 (*P*<0.012; Figure 5). An average of 24% decrease in tumour volumes was detected across all time points. It is conceivable that this effect can be improved by optimising LCL29 treatment protocol, for example, by escalating LCL29 doses, and/or by other SL agents. These are important data showing for the first time that PDT tumour response is promoted by an SL-modulating agent.

This study demonstrates for the first time that Photofrin-PDT has a definitive effect on the SL profile not only in SCCVII cells but also *in vivo*. Specifically, DHceramides are elevated *in vitro* and in tumours after PDT. These novel findings support the notion that the *de novo* SL pathway is a PDT target. Others (Bose *et al*, 1995; Perry *et al*, 2000; Charles *et al*, 2001; Min *et al*, 2007; Senkal *et al*, 2007; Wang *et al*, 2008) and we (Wispriyono *et al*, 2002; Dolgachev *et al*, 2004; Dolgachev *et al*, 2005; Separovic *et al*, 2007; Separovic *et al*, 2008) have shown the involvement of *de novo* SLs in response to anticancer therapeutics. DHceramide synthase 1 (LASS1)/C18-ceramide have been associated with chemotherapy-triggered killing of human head and neck squamous cell carcinomas (Senkal *et al*, 2007). We observed a significant increase in C18:1-DHceramide, the product of DHceramide synthase 1. Our findings in SCCVII cells support the notion that, besides DHceramide synthase 1, other DHceramide synthases, such as DHceramide synthase 2 and DHceramide synthase 4, might be involved in increasing DHceramide levels after PDT. A marked increase in tumour C20-DHceramide further supports the role of DHceramide synthase 2 and DHceramide synthase 4, which have specificity for C20–C26 and C20±2 fatty acyl CoA, respectively (Pewzner-Jung *et al*, 2006; Laviad *et al*, 2008). These findings imply that the accumulation of tumour DHceramide might serve as a biomarker of tumour response to PDT and that targeting the *de novo* ceramide pathway might be a strategy for drug development to advance PDT tumour response.

Another key finding of this study is that co-exposure of SCCVII tumours to Photofrin-PDT and LCL29 leads to enhanced retardation of tumour growth compared with Photofrin-PDT alone. Similarly, LCL124, a structural analogue of LCL29, together with the anticancer agent gemcitabine, effectively inhibits tumour growth of human head and neck squamous cell carcinomas *in vivo* (Senkal *et al*, 2006). Endogenous ceramide levels were not changed after 24 days, that is, at the end of *in vivo* studies after LCL124 or after the combination with gemcitabine (Senkal *et al*, 2006), suggesting no long-term effect of the treatments on ceramide metabolism. Notably, LCL29 evokes a time-dependent increase in total ceramide levels over 24 h in MCF-7 cells (Szulc *et al*, 2006). Selective increases in C16-, C14- and C18-ceramides and decreases in C24- and C24:1-ceramides were observed (Szulc *et al*, 2006). The signature effects of LCL29 alone or in combination with Photofrin-PDT on the SL profile in SCCVII cells and tumours remain to be determined.

In summary, this is the first report demonstrating that following Photofrin-PDT, definitive changes in the SL profile in SCCVII cells and tumours are triggered, and that tumour response is improved after the combined treatment with Photofrin-PDT+ceramide analogue LCL29. Thus, our study validates the therapeutic efficacy of LCL29 in combination with Photofrin-PDT. These findings indicate that ceramide analogues hold the potential of a new class of adjuvants for advancing PDT therapeutic successes.

## Figures and Tables

**Table 1:** Statistical analysis of SL responses to PDT in SCCVII cells

	**Fold increase**	*P-value	**P-value
C14-Cer	3.1	0.154	0.222
C16-Cer	3.7	0.063	0.136
C18-Cer	5.1	0.041	0.112
C18:1-Cer	4.0	0.028	0.104
C20-Cer	6.1	0.046	0.112
**C20:1-Cer**	**7.3**	**0.016**	**0.088**
C22-Cer	4.3	0.146	0.222
C22:1-Cer	6.5	0.127	0.222
C24-Cer	3.4	0.370	0.400
C24:1-Cer	3.7	0.278	0.357
C26:1-Cer	2.5	0.153	0.222
**C14-DHCer**	**10.5**	**0.005**	**0.059**
**C16-DHCer**	**11.8**	**0.003**	**0.059**
C18-DHCer	3.4	0.266	0.357
**C18:1-DHCer**	**9.6**	**0.017**	**0.088**
C20-DHCer	2.4	0.309	0.365
C20:1-DHCer	4.6	0.099	0.197
**C22-DHCer**	**5.3**	**0.007**	**0.059**
C22:1-DHCer	8.9	0.289	0.357
C24-DHCer	5.8	0.047	0.112
C24:1-DHCer	3.3	0.035	0.112
C26:1-DHCer	1.3	0.825	0.858
DHSph	6.3	0.369	0.400
Sphingosine	1.0	0.995	0.995
DHS1P	9.8	0.026	0.104
S1P	6.9	0.145	0.222

C14-Cer=C14-ceramide; C14-DHCer=C14-DHceramide; DHSph=DHsphingosine; DHS1P=DHsphingosine-1-phosphate; S1P=sphingosine-1-phosphate.

After overnight incubation with Photofrin (20 *μ*g ml^−1^), SCCVII cells were exposed to light (0.5 or 1.0 mJ cm^−2^) and incubated at 37^o^C for 4 h before collection for MS analysis. Fold increase refers to an increase in the level of each SL after PDT per 1 mJ cm^−2^ of light. ^*^P-value, nominal *P*-value. ^**^P-value, FDR-adjusted *P*-value. Bold type values indicate statistical significance.
